# To err is human but to persist is diabolical: Toward a theory of interactional policing

**DOI:** 10.3389/fsoc.2024.1369776

**Published:** 2024-05-10

**Authors:** Tanya Stivers, Andrew Chalfoun, Giovanni Rossi

**Affiliations:** Department of Sociology, University of California, Los Angeles, Los Angeles, CA, United States

**Keywords:** conversation analysis, preference, social norm, reproach, violation, social interaction

## Abstract

Social interaction is organized around norms and preferences that guide our construction of actions and our interpretation of those of others, creating a reflexive moral order. Sociological theory suggests two possibilities for the type of moral order that underlies the policing of interactional norm and preference violations: a morality that focuses on the *nature* of violations themselves and a morality that focuses on the *positioning* of actors as they maintain their conduct's comprehensibility, even when they depart from norms and preferences. We find that actors are more likely to reproach interactional violations for which an account is not provided by the transgressor, and that actors weakly reproach or let pass first offenses while more strongly policing violators who persist in bad behavior. Based on these findings, we outline a theory of interactional policing that rests not on the *nature* of the violation but rather on actors' moral *positioning*.

## Introduction

Humans are a highly prosocial, cooperative, and norm-oriented species. Yet our social conventions are routinely violated in everyday interaction. We might do so for cause, for example, by ignoring someone's greeting because we are angry with them. Or we might do so by accident, as when we fail to answer a question because we did not realize it was directed to us. Whatever the reason, actors sometimes depart from expected behaviors. Observers of such departures may respond to the problematic conduct by reproaching the transgressor. At other times, they let violations pass without drawing attention to fault. Using video-recordings of naturally occuring interaction, this paper examines when and how actors respond to each other's problematic conduct, shedding light on the underlying mechanics of interactional policing.

Social norms are the primary conceptual framework that sociologists have relied on to explain moral order. Defined as prescriptions for and prohibitions against particular actions and objectives, norms provide shared criteria for evaluating the conduct of oneself and others (Jasso and Opp, 1997; Horne and Mollborn, 2020). Moreover, they exert moral obligations that go beyond mere habit or convention (Coleman, 1990), creating an alternative motivation to instrumental rationality (Weber, 1947; Elster, 1989).

The salience of a particular norm to a given situation makes relevant the interpretation of others' behavior as complying or departing. This allows for deviant actions to, at times, be sensibly accounted for and rendered mutually intelligible. Thus, norms also provide “resources for understanding,” tools by which actors categorize others' behaviors and recognize themselves to be in particular situations (Heritage, 1984; see also Swidler, 1986). Indeed, the sense-making properties of norms—in allowing actors to experience and organize one another's behavior as normal and routine—may be more fundamental than their regulatory features (Maynard and Heritage, 2023).

Adherence to norms, as well as to other conduct-guiding mechanisms, is encouraged because conformity generally promotes outcomes that are rewarded and rewarding among interactants (Coleman, 1990; Ostrom, 1990). However, in explaining how interactants reproduce norms over time, researchers have focused on sanctions—reactions to violative behavior that seek to punish the actor for failing to conform (Gibbs, 1966; Horne and Cutlip, 2002; Horne and Mollborn, 2020).^1^ Where commitment to norms is in question, the threat of punishment makes departures undesirable by increasing costs (Becker, 1968; Coleman, 1990). Beyond motivating individual compliance, sanctions maintain normative structures by reaffirming the collective moral relevance of operative norms (Parsons, 1937; Durkheim, 1984), thereby distancing the community from “polluting” acts (Douglas, 2005, p. 3–5; see also Garfinkel, 1956). Thus, sanctions not only disincentivize non-compliance but also—and perhaps more consequentially—symbolically reenact the norm's relevance as a feature of shared sense-making.

Despite incentives for norm-conforming behavior and the threat of sanctions for norm-departing behavior, norms are regularly breached, and even widely accepted norms are under-enforced (Horne and Cutlip, 2002). Sanctioning others is rarely rewarded and is itself costly, both because it requires an expenditure of time and effort and because it threatens the enforcer's relationship to both the violator and to observers (Kiyonari and Barclay, 2008; Przepiorka and Liebe, 2016; Kim and Zuckerman Sivan, 2017). While all group members benefit from the enforcement of norms, often it is only sanctioners who bear costs (Fehr and Gächter, 2002). As a result, sanctioning is subject to free rider problems, with individuals relying on others to respond to violations (Oliver, 1980). Consistent with this, in quasi-experimental field studies where confederates systematically violate norms by littering, standing in the wrong place on an escalator, or playing loud music on a train car, researchers find that few observers confront violators (Balafoutas and Nikiforakis, 2012; Przepiorka and Berger, 2016).

Although this literature explains a broad pattern of under-enforcement, we know little about when and how actors *do* enforce shared interactional rules in the course of naturally-occurring everyday interaction. In addressing these questions, we evaluate two main possibilities furnished by contemporary sociological theory for the type of moral order that underlies the policing of interactional norm and preference violations: a morality that focuses on the *nature* of violations themselves and a morality that focuses on the *positioning* of actors as they maintain their conduct's comprehensibility, even when depart from norms and preferences.

Ultimately, we show that actors who make their conduct comprehensible when violating an interactional rule are rarely reproached whereas those who violate rules without regard for others' ability to make sense of their conduct are regularly reproached. Moreover, actors who persist in rule-violating behavior tend to be reproached strongly rather than weakly. We further show that interactional morality constrains how violators design accounts that are acceptable to others, and that the mechanics of interactional policing systematically privileges the position of violations within a trajectory of behavior over the severity of the violation itself. We conclude that the type of morality that underlies interactional policing is rooted not in the *nature* of the violation but in actors' moral *positioning*, where actors are primarily concerned with maintaining the comprehensibility of social behavior.

## Background

In this paper, our point of departure is Goffman's (1983) conception of the *interaction order*, namely, that social interaction constitutes its own domain, governed by a system of conduct-guiding mechanisms that constitute the procedural basis for ongoing joint action (see also Goffman, 1971; Rawls, 1987; Schegloff, 1988). Even in free-flowing conversation, participants attend to and enact a variety of norms that help to maintain mutual intelligibility and promote cooperation. For instance, turn-taking norms help co-interactants avoid overlapping talk, minimize silence, and achieve rapid floor transfer across speakers (Sacks et al., 1974). Norms such as those that mandate responses to actions like greetings and questions facilitate interactional progressivity (Schegloff, 1968). Scholars documenting interactional norms have relied on both the frequency of norm-conforming (vs. norm-departing) conduct as well as orientations by both producers and recipients of conforming/departing conduct. Whereas norm-guided behavior is seamless, departing behaviors are commonly treated as problematic, whether through pauses, laughter, or reproaches such as “I asked you a question” (Schegloff, 1968; Pomerantz, 1984; Zimmermann and West, 1996; Clayman, 2017).

Interaction scholars have described a further level of conduct-guiding mechanisms known as *preferences* (Schegloff, 2007; Pomerantz and Heritage, 2012). In this usage, preferences are not psychological dispositions, nor tendencies driven by instrumental rationality, but rather interactional structures that favor prosocial behaviors, independent of actors' personal inclinations. In line with Goffman's conceptualization, both norms and preferences run through the fabric of conversation, and speakers treat departures from both as problematic (Schegloff, 1968; Heritage, 1984; Pomerantz and Heritage, 2012). As with norm violations, interactants are sensitive to preference violations and display this through such behaviors as delays and mitigations of dispreferred actions.

Consistent with other aspects of social conduct discussed above, in interaction neither norm nor preference violations are consistently reproached by co-participants, and the severity of the reproach—the extent to which it draws attention to the violation and highlights the speaker's responsibility—is variable. However, while consistently emphasizing the moral responsibility of interactants to reproduce shared interactional expectations, existing conceptions of the interaction order lack a theory of interactional policing of norms and preferences. This invites our central empirical questions: When and how do actors reproach others for interactional violations? Two lines of theory related to morality and social conduct offer potential avenues forward.

One explanation for reproaching behavior, implied by divergent theoretical perspectives, relates to the *nature* of the violation as constituting a moral failing. A variety of social scientific scholars of morality conceptualize actions (e.g., interrupting or lying) as having isolable properties that reflect on the character of the actor (e.g., as a bully or a phony). For instance, Tavory (2011) argues that “moral actions” are those that define the actor in a particular, typified way that has transsituational implications, and can be expected to draw a standardized emotional response. Similarly, Stets and Carter (2011, 2012) argue that individuals with high perceptions of their own morality are more likely to engage in conduct that abides by normative expectations. These approaches imply that individuals will treat negative actions as having a stable impact on moral worth that is relatively independent of the environment in which the action occurs (Lemert, 1951), and that actors evaluate concrete actions on the basis of general principles drawn from a shared worldview or cultural repertoire (Swidler, 1986; see Boltanski and Thévenot, 2006).

Such an emphasis on the object of moral evaluation—relatively disembedded from the environment in which it takes place—finds expression in the emphasis on self-reported assessment within much of the social psychology of morality (see Abend, 2013 for a critique; Skitka et al., 2021). This perspective tends to imply that reactions to a violation are a function of the *nature* of the violation itself, with stronger violations attracting a sharper emotional reaction (Tavory, 2011, p. 282, 283). That such a balance between the nature of the violation and the strength of the reaction exists is further suggested by the widespread belief that the “punishment should fit the crime” (Hamilton and Rytina, 1980) and by evidence that individuals can react negatively to those who sanction minor offenses (Eriksson et al., 2017).

Approaches that emphasize individual actions can be juxtaposed with an alternative tack that shifts away from the transsituational properties of moral conduct and attends to the practices by which interactants render their conduct comprehensible (Garfinkel, 1967). This stream of work moves the nature of the violation to the background and instead foregrounds the context of ongoing activities and projects, linking our conduct to prior commitments and future aims that give meaning to our actions (Emirbayer and Mische, 1998; Mische, 2014). On this view, violations do not stand in isolation, but rather are situated in a larger trajectory, including the individual's past behavior alongside expectations about the future (MacIntyre, 1981). Where this sense of larger trajectory breaks down, typical moral calculations become unsustainable and are subject to radical revision (Bittner, 1967). We refer to both the situated character of evaluation and the work that goes into rendering actions conceivable within broader trajectories as the moral *positioning* of the actor (Harré and Langenhove, 1991), which we will now unpack more fully.

Sociologists in the ethnomethodological tradition have argued that there is a strong orientation to the relevance of maintaining mutual intelligibility in interaction. However, early ethnomethodologists departed from then-dominant cultural models (Parsons, 1961; Geertz, 1973) that treated intelligibility as resulting from the successful application of an internalized cultural system or worldview to a particular object. Instead, they emphasized the procedures—“methods”—that interactants employ within an unfolding context. In managing emergent contingencies, interactants seek to make sense of their encounters with others by fitting actions into a coherent trajectory (Garfinkel, 1967; Heritage, 1984; Zimmermann and West, 1996). Individuals are thus continuously evaluated and held accountable by others on the basis of observable features of their conduct and of the local context in which those actions are situated (Garfinkel, 1967). Drawing on these evaluations, interactants form judgements about what others are doing and respond accordingly. Actors may shape others' interpretations by characterizing their conduct or the situation with *accounts*, which “explain untoward behavior and bridge the gap between actions and expectations” (Scott and Lyman, 1968, p. 46). In producing an account, actors work to retrospectively position themselves within a coherent trajectory and to present their actions either as exceptions or as falling within accepted patterns of conduct despite appearances to the contrary (see also Vaisey, 2009; Winchester and Green, 2019). However, an actor's range of acceptable accounts is limited by the interpretive practices of co-participants, who may reject an account for problematic conduct as insufficient (Scott and Lyman, 1968, p. 54). Along these lines, accounts centered on motives or incompetence are treated as inferior to accounts articulating a competing obligation (Goffman, 1971, p. 110–112; Drew, 1984; Heritage, 1984, p. 270–272). The ethnomethodological tradition offers a distinctive approach to understanding moral obligations. Rather than focusing on the commission of a violation, in and of itself, as the basis for others' reactions, this research emphasizes the violation's location within a sequence of actions understood with reference to a mutually accessible goal. The basis for reproaching others and thus treating their conduct as morally problematic then, is the failure to render an action intersubjectively comprehensible.

Alongside work on comprehensibility, an emphasis on moral positioning is visible in research concerned with the consequences of repeated conformity or deviance on an individuals' reputation. Much of the sociology of morality—including work that treats the individual action as a moral object—has argued that conformity to or deviation from shared expectations is reflective on the self, so violations of normative conduct have negative implications for one's moral character (Tavory, 2011) and encourage individuals to act in ways that confirm their and others' perceptions of who they are (Becker, 1963; Stets and Carter, 2012). Generalized expectations about the kinds of projects that an individual typically engages in—along with the means that the individual is expected to deploy to forward those projects—constitute a *reputation* (Fine, 2019). One's reputation is subject to ongoing revision and reevaluation, and sociologists have described how reputations can be damaged through processes of penalization, degradation, and demonization (Lemert, 1951; Garfinkel, 1956; Goffman, 1961; Ducharme and Fine, 1995). Such processes identify the target's whole person with some deviation, rendering previous virtuous actions irrelevant to estimations of character.

However, while a reputation can be conceptualized as an *outcome* of one's previous performance, it may also be conceived as contributing to action ascription itself. In this latter sense, which is not always clearly distinguished from the former, a reputation is not merely the ossification of repeated actions. Rather, a person's reputation shapes the action that is being performed, so the degree of violation, and even whether or not a violation has occurred, can critically depend on *who* performs the action. For example, in his study of stand-up comedians, Reilly (2018) argues that accusations of joke theft are only loosely related to the similarity of a joke to others' routines. Instead, the determining factor is the comedian's reputation among other performers, with individuals who are perceived as authentically committed to group norms being protected. While in Reilly's case a positive reputation was protective, the reverse may also be true. When a prolonged disjuncture between public commitments and private actions is revealed, a good reputation may trigger heightened sanctions as punishment for apparent duplicity (Adut, 2004; Bartley and Child, 2014). By the same token, repeat offenses are generally treated more harshly than single actions, since the latter can be written off as temporary deviations from an individual's normal practices and dispositions (Becker, 1963; Dana, 2001).

Evidence from interaction suggests that a violation's location within an unfolding sequence, including its proximity to similar violations, is critical to the response it receives. For example, interactants use specialized practices, such as multiple sayings (e.g., “No no no” [Stivers, 2004]), to handle situations where others are unnecessarily persisting in a course of action; and with children, persistence in unwarranted requests leads to escalations of rejection (Wootton, 1981). Further, interactants may resort to increasingly strong tactics to gain another's compliance in the face of persistent problematic behavior (Kidwell, 2006).

In this study, our focus is on interactional norms and preferences that operate when people produce responsive actions (e.g., answering a question, granting a request, agreeing or disagreeing with an assessment). We ask when and how departures from such interactional norms and preferences are reproached. Further, within the set of cases where an actor does reproach another, we ask what explains the use of a stronger or weaker type of reproach. In addressing these questions, we make three main theoretical interventions. First, we outline a theory of interactional policing grounded in the comprehensibility and persistence of social conduct. As part of this, we offer a taxonomy of practices that participants who witness a departure use to reproach another's behavior. While previous work on norm enforcement has focused almost exclusively on sanctioning, this study situates sanctions within a broader ecology of available responses—which we collectively term “reproaches.” Second, we intervene in theories of morality and normativity by clarifying the relationship between individual actions as objects of moral evaluation and the contexts in which they are produced. We show that, in policing the interaction order, participants systematically privilege the position of violations within a trajectory of behavior over the severity of the violation itself. Third, we extend the literature on accountability and the practice of accounting for one's conduct by deepening our understanding of what makes accounts for departures acceptable to others.

## Data and methods

To investigate the policing of interactional rules, we examined audio and video recordings of naturally occurring social interactions, using conversation analysis (CA) as our primary method (Sidnell and Stivers, 2012), with insights from abductive analysis to bring theoretical reflections into our analysis early to facilitate generative theory building (Tavory and Timmermans, 2014). We approach social interaction as an ordered domain with its own structures (Goffman, 1983; Heritage, 1984). As such, we did not simply look for times when individuals seemed to do something untoward but rather we began by identifying a structural context that creates a systematic opportunity for participants to conform to, or depart from, interactional norms and preferences.

Specifically, we identified sequences of paired actions such as greeting sequences (A: *Hi!* B: *Hey*.), summons-answer sequences (A: *Bella?* B: *Yeah…*), request-response sequences (A: *Can you hand me the hammer?* B: *Here ya go*./hands over the hammer); or story tellings and their uptake. In a subset of these sequences, the respondent's behavior involved a violation of an interactional norm or preference such as not returning a greeting, not responding to a summons, or refusing a request—whether or not there was an explicit orientation to the violation. Our focus on this subset, constrained by sequential context, helped to provide a natural control for the range of rule-conforming/non-conforming behaviors under analysis. Following conversation analytic principles, our goal was to examine how robust the patterns of behavior were across situations and individuals. Thus, while constraining the sequential context, we strove to represent a wide variety of settings and activities in order to maximize diversity in the data, ensuring that the patterns and practices we identified transcend individuals, interactions, or other ways of categorizing the data (e.g., by gender, race, or other social categories).

As discussed in the previous section, departures from interactional norms and preferences are uncommon. Therefore, we gathered these cases opportunistically across existing video and audio corpora of over 40 American English and Italian informal conversations, supplementing this with additional cases from outside the main corpora (e.g., from a talk show). These corpora were originally collected primarily by two of the co-authors (Stivers and Rossi) by soliciting consent from participants and then setting up a camera in their home or workplace, which would record continuously for an agreed upon period of time, commonly 45–60 min, but up to a few hours for longer activities. In examining these corpora, we first looked for similarities and differences in the way that speakers of American English and Italian behaved in interactions involving a norm violation. The overwhelming similarities led us to build a joint collection, following the approach of cross-linguistic “co-investigations” (Lerner and Takagi, 1999).

Initial observations on a small, heterogeneous set of norm violations led us to restrict our collection to departures in responsive (rather than initiating) sequential position, and to expand the collection to include departures from interactional preferences as well as norms. In conversation, multiple norms and preferences may be relevant in a given context (e.g., in a question-response sequence, the question makes relevant a response; an answer response is preferred over a non-answer response; and a confirming answer is preferred over a disconfirming one). For our purposes, a departure from any of the relevant norms or preferences constitutes a departure that interactants might reproach. In keeping with our focus on the interaction order (Goffman, 1983), we excluded cases where the primary departure was not related to the structure of interaction (such as when children violated table manners or family rules, or students violated university ethics).

In the next phase of collection building, and borrowing from abductive analysis (Tavory and Timmermans, 2014), we worked to add departures that specifically involved a “reproach” while still drawing on a wide variety of unscripted interactional contexts. This meant that we expanded the collection with additional instances from our main corpora of ordinary social interaction as well as instances from other settings (e.g., televised interactions), as in the case we examine from a talk show.

In the final phase of collection building, we targeted a range of diverse types of departures from competitive overlaps to failures to align to a story to marked confirmations to non-selected next speakers taking the next turn. This helped to provide sufficient breadth in our collection beyond the most common types of departures (e.g., failures to respond and dispreferred responses). We stopped building the collection once we reached saturation and were no longer gaining insight from additional instances of departures.

We did not collect every departure in our corpora, so we cannot make claims about the basic frequency of reproaches in interaction. Rather, our goal was to have a sufficient number of departures with and without reproaches to explore the question of what kinds of departures attract reproaches as well as to examine how speakers design reproaches. In total, we identified 103 departures (62 in American English; 41 in Italian) across 44 interactions (22 in American English; 22 in Italian). We found no substantial differences in how speakers of American English and Italian handled interactional departures. Table 1 offers an overview of the four types of reproaches that we identified in our collection along with examples. Each of these types has a basis in existing literature but, as we explain in what follows, they represent distinct interactional mechanisms and can be conceptualized as increasing in strength.

**Table 1:** Types of reproach to interactional departures.

**Reproach type**	**Examples**	***N***
Pursues	“Your turn” “Come on”	23
Requests confirmation	“You don't want a beverage?” “Of course?” “Really?	4
Challenges the action/inaction	“Why not?” “Didju hear me?” “Yes!” (*(contradicting other)*)	16
Sanctions	“Answer the damn question” “Stop talking”	19

When there is a violation, as when a response is not forthcoming or is dispreferred (e.g., a refusal), an interlocutor may nudge for a rule-conforming response with a *pursuit* (Pomerantz, 1984; Jefferson et al., 1987), which treats the actor as having not yet produced the optimal behavior but as having a still in-progress action trajectory. Alternatively, the interlocutor may *request confirmation* of the departing behavior (Schegloff, 1979), thus providing the actor with an opportunity to change tack. A third type of reproach involves the use of a *challenge* where the interlocutor treats another's behavior as problematic by soliciting an explanation (e.g., Bolden and Robinson, 2011) or by contradicting the problematic action with a counter-response (e.g., responding to a “No” with “Yes!”). Though distinct, these types of reproach still allow for the violator to modify or justify the behavior. Finally, with a *sanction*, the interlocutor explicitly admonishes the actor's violation as problematic, treating the response *as* a violation (e.g., Schegloff, 1968; Heritage, 1984), often indicating what s/he should have done or what s/he should not have done.

All of these reproach types are on-record orientations to violations and make relevant responses by interlocutors. We distinguish reproaches from off-record orientations to a violation such as silences, laughter, or otherwise moving on in the conversation. While the latter also constitute ways for participants to convey that there has been a violation, they do not alter the course of the interaction to either thematize or push back on what an actor has done. We do not consider these indirect ways of dealing with a violation as *reproaches* because they are neither concerned with a revision of the violation nor with delaying conversational progressivity to address it. Rather, we conceptualize reproaches as more overt ways of treating an actor's behavior as a violation that alter the course of the interaction and make the violation a source of repair or trouble. We over-sampled these in order to examine how reproaches were designed.

Consistent with conversation analytic methods, our analysis began by examining our collection for similarities and differences, looking for data-internal patterns in the association between attributes of the departure and the response to it (or lack thereof). Our goal was to explain whether and how a departure was reproached that would hold up independent of other aspects of the context. We then used structured coding (Tavory and Timmermans, 2014) to test the robustness of the patterns we identified across the data and to make sure that particular dyads/triads were not driving the results. We also coded whether the departure was from an interactional norm or preference to explore whether norm violations are treated differently than preference violations.

Consistent with previous treatments (Schegloff, 2007; Stivers, 2022), we conceptualize interactional norms as stronger than interactional preferences, one reason being that preferences are nested within norms. To illustrate this, consider question-response sequences. If Amy looks at Bernie and asks “Is the birthday party on for tonight”, her question invokes the norm that a response is due to complete the sequence (Schegloff, 1968; Schegloff and Sacks, 1973). Moreover, while Amy produces her question, a concurrent turn-taking norm dictates that Bernie should not overlap her talk nor compete with it. As these norms are followed, a series of preferences become relevant including the preference for the selected speaker to speak next (Sacks et al., 1974); the preference for an answer over a non-answer response (Clayman, 2002; Stivers and Robinson, 2006); the preference for confirmation (e.g., “Yes”) over disconfirmation (e.g., “No”) (Heritage, 1984); and the preference for unmarked interjections over other forms of confirming answer types (Raymond, 2003; Stivers, 2022). The relative strength of interactional norms over preferences is further reflected in perceptions of deviant communicative behavior in autistic individuals, for example, in the salience of failing to respond to another person altogether compared to failing to show sufficient empathy in a response (Schopler and Mesibov, 1985).

Once data were coded, we used inferential statistics in the form of generalized linear mixed models to test quantitative relationships between violations and reproaches, and between persistence of violations and reproaches. In doing so, we also controlled for the particular episode of interaction in which participants were engaged (see Appendix B for details).

In what follows, we begin with the question of what explains the presence or absence of a reproach to a departure. We consider both the type of departure and the way that the departure is produced in light of the theories reviewed in the Background. We then discuss the forms and varying strength that reproaches take and ask what explains the strength of an interactant's reproach to another's departure.

## Results

As with other aspects of social life, in social interaction, individuals monitor one another's behavior, but only sometimes reproach another for departures from rule-conforming conduct. Given the various explanations for not enforcing interactional norms and preferences discussed above, our overarching empirical questions are when and how do people police violations of rule-conforming conduct. We begin with the “when” and show that speakers are more likely to reproach another when their departure is not accounted for and is therefore hard to fit within a sequence of appropriate actions. We utilize our empirical findings to speak to alternative theories of morality and normativity, addressing competing predictions that these theories make about what drives the policing of interactional conduct. We also intervene in the literature on reputation by explaining its mechanics at the micro-level of interaction.

### The comprehensibility of action

In this section, we argue that interactional policing in the form of reproaches consistently occurs when actors fail to show an orientation to their accountability for rule-departing conduct, whether from interactional norms or preferences, usually by offering an explanation. In providing accounts, we suggest that actors orient to the morality at issue in violating interactional norms and preferences not with respect to the severity of their violation but rather regarding the comprehensibility of their actions.

As a first prong of evidence, consider that when actors do not provide accounts for rule-departing conduct, they are consistently reproached. In (1), Ally and her boyfriend Brad are in a car. Brad is driving while Ally is in the passenger seat. Ally is estimating their likely arrival time and is in the middle of a unit of talk when Brad turns on the radio (line 5). The violation concerns competing with someone's in-progress talk. While this commonly takes the form of two people talking simultaneously, the violation here is that, as Ally revises her estimate of their arrival time, Brad switches from music to talk radio in the middle of Ally's speaking (lines 9/10). Then, as she continues speaking in competition with the radio, Brad turns it up (lines 12–14). The volume by this point is so loud that it is hard to hear Ally in the recording, so it is highly competitive with her talk.


(1) MD (See Appendix A for transcription conventions)
1  AL: I say:,
2   (2.5)
3 AL: Ahhh I was gonna say eleven
4     [^°^thirty^°^ = I say eleven twenty,
5 BR: [((turns on the radio))
6 AL: You say eleven third-
7    No I say twelv-(0.8)
8    N[o. I say eleven twenty.
9  BR: [((BR switches to news))
10 RA: [Massachusetts senator
11    Ted Kennedy=
12 RA: = M[OST OF THE TIME,
13 AL:    [Eleven twenty.
14 BR:    [((Turns volume up high))
15    (0.6) ((Radio stays loud))
16 AL: HEY:: ((AL looks at radio
17    then back to BR))
18 BR: I'd like to hear the news
19    just to [distract me fro-
20 AL:    [So loud?
21 BR: Ye(h)ah ((yawning))
22 AL: hck hck hem ((throat clearing))
23 BR: Just to distract me from?
24   (.)
25 BR: (^°^Okay^°^)


After monitoring Brad's behavior for approximately half of a second (line 15), Ally sanctions it with “HEY::” while looking from the radio to Brad. Although this sanction does not explicate what the violation is (Schegloff, 1968), its positioning makes clear that it is focused on the competing radio. In the tussle that follows, Brad shows that he understands the radio as the problem (lines 18/19), but he treats it as having the radio on at all, while Ally—without letting him complete his turn in lines 18/19—treats the problem as one of how loud the radio was (line 20).

Notice also that the reproach only comes once it is clear that Brad intends to keep the radio loud, and that his behavior is thus not attributable to an accident. Specifically, Ally withholds the reproach across lines 9 and 12. In line 9, the radio is turned up but she initially continues to speak in competition with it. Then, during the silence at line 12, she monitors Brad and the radio for roughly half a second before reproaching him for obstructing her talk. In sum, only when it is apparent that Brad is not going to remedy his behavior, nor provide an account, does Ally reproach him. Thus, she treats the main offense as not simply the turning on or up of the radio but rather the failure to maintain his action's comprehensibility.

Extract (2) provides another example where there is a departure without an account. This time the departure is a failure to respond to a question. Here, friends are meeting at Ada's place. Just prior to the extract, they were discussing dogs barking, which has been an issue in Ada's neighborhood. Touched off by this, Bruna asks Ada how things are going with her neighbors (lines 4–5/7). Ada refuses to respond, saying: *Look mm Ask me another question* (“Vara: mm Fame 'n altra domanda.”). After acknowledging Ada's refusal to respond with a loud *RIGHT* (“SÌ.”), Bruna goes on to challenge Ada (lines 12–15 and 17–18), using a large jump to high pitch (↑↑) and seeking an account for the violation (Bolden and Robinson, 2011).


(2) DopoProve09-2_483078
1 MI: Ha un codice fiscale il cane?
   **Does the dog have a social**
   **security number**
2    (.)
3 MI: ( [ ) (co]municarsi.)
   **(    ) (communicate)**
4  BR:    [Ma e come st-]
   **And how ar-**
5   Come state [andando con i]=
    how stay-2PL go-GER with the
   **How are things going with the**
6 MA:   [ ((laughs))  ]
7 BR: =vi[cini.
   **neighbors**
8 MI:    [((laughs)) 
9 AD: Vara: mm Fame 'n
    look-IMP.2SG mm ask-IMP.2SG=1SG.DAT one
   **Loo:k mm Ask me**
10     altra dom[anda. ((shakes head))
 other question
   **another question**
11 BR:   [SÌ.
 yes
   **RIGHT**
12    ↑↑Ma senti com'ela che
    but hear-IMP.2SG how=be.3SG=SCL COMP
   **But listen how come do I**
13   co- devo continuare
    co- must-1SG continue-INF
   **co- do I have to continue**
14     a farte £altre
   **asking you different**
15    do[mande o con]tinua(hh)re=
   **questions or continue**
16 AD:   [((laughs)) ]
17 BR: =a fa(hh)r- .hh a cambiar
   **d- .hh keep changing**
18     disco:£rsi.
   **subjects**


In contrast to the two violations we just examined, in cases where actors do offer accounts, reproaches are uncommon. Extract (3) is from a conversation at a sorority house. Trish asks Nicki whether she went to an event the night before (line 1). After a delay (line 2), Nicki disconfirms the question and includes the account “I was writing uh paper.” Trish does not reproach Nicki and instead goes on to volunteer information on the event.


(3) SB2: 20:50
1 TRI: Oh did you go last night Nicole?,
2     (1.2)
3 NIC: No I was writing uh paper.
4 TRI: Oo::


The next extract gives us two violations in the same sequence of interaction: for the first violation, an account is not given but is readily inferable; for the second violation, an account is neither given nor inferable. As we will see, the interlocutor responds differently to the two violations. In (4), several people are gathered in an apparently closed upholstery shop where at least one of them works. In line 3, Mike asks *What's the difference between a hassock and an ottoman*.^2^ This is an audio recording of face-to-face interaction, so it is not clear whether Mike is looking at Joe at that point. Regardless, the subsequent address term “Joe,” (line 8) disambiguates the selected next speaker. Vic starts to answer before the address term is fully articulated. This provides a possible explanation for why Vic might respond to a question that selected Joe: the identity of the selected next speaker was unclear when Vic began to respond. In this context, Mike disregards Vic's answer, re-addressing Joe (line 6). Mike then redoes the question in lines 11/13, asking the question “for another first time” (Schegloff, 1992), without reproaching Vic.


(4) Upholstery Shop 91:41-91:47
1  VI: A ha:-:ssock, (.) en o:ttoman_
2   [a ha-assock,
3  MI: [What'sa difference between
4   uh hassock en 'n ottoman=
5   {=J[oe.
6  VI:   [Uh:, You put=cho feet
7   on [(it.)
8  MI:    **[Joe,**
9   (0.7)
10 CA: (Mm [hm,)
11 MI:   **[Whatsa d[iffrence b'tween=**
12 VI:        [No difference.
13 MI:  **=uh hassock en 'n ottoman.**
14 JO: [I think it's the shape].
15 VI: [De  diffrence  between] uh sofa
16     'n uh cou[ch. ((louder))
17 CA:       [A hassock
18     [is square like that one  you]
19 MI: **[Waidaminnit I didn' ask you,]**
20   [did befaw.
21 VI: [Oh:. (bah[:.)
22 JO:      [I think it's the
23   shape.


Vic answers in overlap with Mike re-asking the question, but at this point his behavior is more problematic: he not only knows that Joe has been selected as the next speaker but also competes for the floor, first with Mike and then by expanding his answer in overlap with Joe's talk (lines 14–16). This time, Mike reproaches him: “Waidaminnit I didn' ask you,”. This explicitly treats Vic's response as inappropriate.

Critical here is that, when there was an available account for Vic's departure (that Vic might have thought the question was for him), Mike did not police Vic. However, as soon as there is no available account, he does. This suggests that the account matters for interactional policing. Note too that the nature of the second violation is essentially the same as the first, but it is treated very differently. This again lends support to the idea that interactional morality is not grounded in the *nature* of violations but rather in the moral *positioning* of interactants through maintaining comprehensibility of their actions within larger trajectories.

Across the dataset, we see that, of the departures for which an explicit account was provided by the actor when the departure was done, only 21% (*n* = 7/34) were reproached. Conversely, when actors offered no account at the time of their departure, interlocutors reproached them 80% (*n* = 55/69) of the time, a significant difference. The odds of an unexplained departure being reproached increased by nearly 16 times relative to an accounted-for departure (OR 15.6, 95% CI 3.67–64.74, *p* < 0.001) (see Appendix B for details). As noted in the Background, there are multiple reasons for a co-interactant *not* to issue a reproach in the face of a departure. Moreover, a reproach is never interactionally mandated as a conditionally relevant next action (Schegloff, 2007), and thus cannot be said to be “missing.” What matters here is the interactional policing that is present in the form of reproaches that *are* issued relative to the presence or absence of an account for the departure.

Further support for the idea that the *nature* of the violation is not the overriding issue for interactional policing is found by comparing reactions to norm departures (which can be conceptualized as more egregious, see Data and Methods) with reactions to preference departures. While norm departures were reproached somewhat more often than preferences departures (75%, *n* = 15/20 vs. 57%, *n* = 47/83), this difference is not statistically significant (*p* = 0.144) (see Appendix B for details). This suggests, once again, that the severity of the violation itself is not the primary underlying theoretical explanation for reproaching.

Another major prong of evidence for the importance of maintaining the comprehensibility of actors' conduct is that, with certain kinds of reproaches, interlocutors solicit an account, thus treating it not only as absent but also as relevant to the interactional policing and interpretation of others' conduct. An example of this is found in (5), where the interaction revolves around the dispreferred response of declining an offer. At first glance, it might seem that accepting or declining an offer is simply a matter of personal choice—albeit with different social repercussions (Heritage, 1984). Yet we find that here, too, departing from the preference for acceptance is subject to reproach. In (5), housemates Lance, Gio, and Judy are cooking together. Gio offers “a beverage” to Lance and Judy, likely using the term “beverage” to indicate an alcoholic drink. After a silence, Lance turns down the offer with an unusual disconfirmation form (“I need not.”) that is matched to the use of “need” in Gio's offer.


(5) HM
1  GI: Alright. Who needs a
2   beverage.=hh
3   (0.5)
4  LA: I need not.
5   (0.2)
6  GI: You don't want uh beverage?
7  LA: |NO!|
8   (.)
9  GI: Okay.
10  (.)
11 JU: Why not?,
12  (0.4)
13 GI: h(h)h[h
14 LA:   [Do I  <need> to
15  have uh beverage?,
16 JU: Yes[::,
17 GI:  [You don't
18  [n:eed to have uh beverage_
19 LA: [(°   °)    
20 GI: I'm just wondering if you
21  want one.


Both Gio and Judy treat Lance's dispreferred response as problematic and issue reproaches. Gio's request for confirmation (“You don't want uh beverage?” line 6) constitutes a reproach by treating the response Lance has just provided as not definitive, and by further implying a possible contrast between “need” and “want.” Asking for confirmation of something just stated is a common way of soliciting an account for the behavior (Raymond and Stivers, 2016). Although Gio subsequently accepts Lance's reconfirmation (line 9), Judy goes on to reproach Lance with a challenge (“Why not?,” line 11). Lance resists this as well, but Judy holds firm (line 16).

In sum, here we see first a request for confirmation inviting an account (Raymond and Stivers, 2016) and then, when that fails, a direct request for an account (Bolden and Robinson, 2011) by two different participants. Although Lance does not provide an account to either effort, the point is that, when accounts are lacking, interlocutors solicit them, thus specifically treating the account as missing and important to the comprehensibility of the actor's conduct.

We see this pattern again in (6), where Jill proposes a walk to her husband Chan, which he declines. Chan designs his response as dispreferred through silence and a quiet delivery (noted with the degree signs ^°°^), but provides no account for it (lines 2–3). A moment later, Jill challenges Chan with a request for an account (line 5), which he then provides (line 7), though this leads to other issues (line 9).


(6) CL (at the end of dinner after a lapse)
1 JIL: °You wanna° walk tonight?,
2   (0.4)
3 CHA: °Mh mm.°
4   (0.8)
5 JIL: **Why ^no:t.**
6   (0.5)
7 CHA: >I have to< fix a computer.
8   (1.1)
9 JIL: It's y^our f^au:lt;


Finally, in (7), four fraternity friends are socializing in anticipation of attending a party together. Jan recently told a story in which he reported that he readily told his parents that he had run from the police after getting tangled in a barbed-wire fence as a teenager. Tex was a high-school friend of Jan's, so he knows Jan's parents. Justin is a college friend who met Jan's mom when they visited Jan's house but, as he states in line 1, did not meet Jan's dad. Tex sings Jan's dad's praises (lines 5–7) and then claims “That's Kramer.” (line 9) with a smile (noted with the pound sign £), adding a slight chuckle at the end.


(7) FG 6.30
1  JS: I never met your d\underline{a}d but (.)
2   >°(we stayed at your
3   [house   )° <
4  TX: [Humpf
5  TX: (£He used=tuh hafta fight:
6   mo:m by uh- trillion folds.
7   =[huh)
8  JN:  [Yes
9  TX: £That's Kramer.=hmh
10 JN: I- hh if you
11   me[et 'im-
12 JS:   [Your dad's name's Kra:mer?,
13 JS:   [((gaze to Jan))
14 JN: Yeah of course.
15 JS: **Huhuhuhuhuh of course?,**
16   Huhuhu[huhuhuh
17 JN:    [He's uh legend.
18   HE's UH FUCKIN' LEGend (     ,)
19 JS: **Wh(h)y i(h)s i(h)t of**
20   **c[ou(h)(h)rse.**
21 JN   [Je::sus. .hh huh
22   (.)
23 JN: I mean if you knew my dad you'd
24   know he's Kramer. I mean_
25 TX: God. (0.5) That man. (.) I tell
26   you. (1.2)
27   >Kramer's picked me up numerous
28   times. <
29 JN: °hh hh°
30 TX: and saved my life.


Soon after Jan reveals that his dad's name is Kramer, Justin questions him with “Your dad's name's Kra:mer?,” (line 12). The name may be hard to believe for Justin not only because it is unusual but also because, at this time, the popular TV show *Seinfeld* was running, and *Kramer* was a main character. In response, Jan confirms that his dad's name is indeed Kramer (line 14). However, rather than confirming with an unmarked “Yeah,” he pushes back on Justin's questioning of him by adding the upgrade “Of course”—a preference departure (Stivers, 2022). The departure is minimal in the sense that Jan's response is still a confirming answer to the question, but it relies on a marked interjection that is used in very restricted circumstances.

Jan gives no account for this departure, and Justin reproaches him with a request for confirmation that invites an account (line 15). A moment later, after the request for confirmation fails to secure a satisfactory account, Justin challenges Jan with a direct request for an account (lines 19/20), ratcheting up his treatment of Jan's conduct as not comprehensible. The development of the reproach sequence from a request for confirmation to a challenge is similar to what we saw in (5). In (6), Jan ultimately provides an account (line 1), which is later expanded by Tex. What this case shows, once again, is that reproach practices including requests for confirmation and challenges treat accounts as missing but necessary to make the prior speaker's conduct comprehensible.

That accounts point to comprehensibility as the primary moral issue in the policing of interactional norms and preferences is further underscored by the fact that actors sometimes account for a violation *after* the fact, that is, after an interlocutor's reproach has indicated that their earlier departure was not understandable. Interestingly, we did not observe apologies in these contexts (e.g., “I'm sorry”, “My bad”), which would instead point to the violation itself, or to its severity, as the primary moral issue. Of the 55 cases where the violator did not provide an account at the time of the departure, and the departure was then reproached, the violator continued by offering an account after the reproach in one third of cases (*n* = 18). For instance, in (1), although Brad turns on the radio and then turns up the volume quite high without any account, after Ally sanctions him for his behavior, he provides the account that he wanted to distract himself (lines 18/19).

Another illustration of the relevance of accounts is given in (8), a case where the violator accounts after the fact. Sofia and her boyfriend Furio are baking cookies for Christmas at his house. In that context, she asks if he would come with her to gut and clean sardines for her family's dinner that afternoon. In response, he first demurs (lines 8–14); then assesses this task ironically as *Super fun* (“Stra divertente.”); and ultimately refuses the request with *Also no* (“Anche no,”). There is initially no account provided for his refusal, but after Sofia provides an ironic *Thank you* (“£Gra(h)zie.£”) that reproaches him, he explains: *I believe I have stuff to do this afternoon* (“mi sa che ho da fare °questo pomeriggio_°”).


(8a) BiscottiMattina01_1226190
1  SF: Io oggi pomeriggio devo andare
   **This afternoon I have to go**
2   a casa sai?
   **home you know?**
3   (1.2)
4  FR: Perché?
   **Why**
5  SF: Perché: dobbiamo pulire i
   **Because we have to clean the**
6  pescetti.
   **fingerlings**
7  (0.6)
8  FR: I pescetti?
   **The fingerlings?**
9  (0.4)
10 SF: Le sarde.
   **The sardines**
11  (0.4)
12 SF: Dobbiamo aprirle sbudellarle e
   **We have to open, gut, and**
13  lavarle.
   **clean them**
14  (1.1)
15 SF: °Capi[sci_°
   **Do you see**
16 FR:  [Stra divertente.
         **Super fun**
17 SF: Vuo- vuoi
  want-2SG want-2SG
   **Wou- would you like**
18  aiutarci che ne abbiamo
  help-INF=1PL.ACC/DAT CONN PTV have-1PL
   **to help us since we have**
19  tipo(hh) ce(hh)nto da fare,
   like hundred to do-INF
   **like a hundred to do?**
20  (0.7)
21 FR: Ma:_
  but
   **Well:**
22  (0.5)
23 FR: Anche no,
  also no}
   **Also no**
24  (0.4)
25 SF: £Gra(h)zie.£
   **£Thank you£**
26  (0.2)
27 FR: No non ↑é quello_
  no not be.3SG that}
   **No it's not that --**
28  é che: mi sa che
   be.3SG COMP 1SG.DAT feel-3SG COMP
   **It's that I believe**
29     ho da fare
   have-1SG to do-INF
   **I have stuff to do**
30     °questo pomeriggio_°
   this afternoon
   **this afternoon**


As it turns out, the post-positioned account is not effective for reasons we will describe below, but the main point here is again that participants orient to comprehensibility as the primary moral issue with departures.

In this section, we have shown that participants treat departures from interactional norms and preferences as accountable. First, actors typically provide unsolicited accounts at the moment of the departure. Second, in the face of a reproach following an unexplained violation, actors subsequently account for it, either in response to a solicitation or at their own initiative. These patterns suggest that the moral underpinnings of interactional policing focus not on violations themselves but on the interpretability of social action.

### Accountability vs. comprehensibility

At first glance, it might seem perplexing that an individual could escape reproach with a simple explanation for a violation. Why does that work? In this section, we unpack what it is about providing an account that wards off reproaches and what kind of an accounts legitimately function in this way. Through tackling the question of when departures are reproached and how, we aim to address the theoretical puzzle of what drives the policing of interactional conduct. An analysis of the design of the accounts that actors provide for their departures lends additional support for the claim that, rather than being primarily concerned with the commission of violations *per se*, the moral imperative for interactants is to maintain the interpretability of conduct.

As reported earlier, interlocutors reproached rule-departing actors only 21% (*n* = 7/34) of the time when actors provided an account for their departure. Across the 79% (*n* = 27/34) of accounts that were accepted, two components of their design consistently emerge. First, the accounts typically involve an *external* rather than an internal explanation. While wants, needs, and feelings are *internal* to the individual who experiences them, appointments, work obligations, prior commitments to other people, or diets are treated as *external* circumstances that exert pressure on an individual. Second, the accounts that were accepted typically involve *specific* rather than *vague* explanations. These components suggest that it is not simply the presence of any account that makes the actor's conduct comprehensible but the presence of an account that offers insight into the external and specific constraints that led to an interactant departing from a norm or preference.

This is exemplified if we return to (8a). Furio's account, *I believe I have stuff to do this afternoon* (lines 23–24), is vague with respect to both what he is otherwise committed and when. The account is also uncertain (*I believe/feel)*, and unclear as to how it constrains Furio's ability to help Sofia. The account is external but only superficially so since he does not invoke an outside origin of the “stuff” he has to do, such as an appointment or prior commitment.^^3^

It is on the basis of these weaknesses in his account that Sofia takes issue with Furio. In (8b), continuing from (8a), we see that she questions his account, first by requesting confirmation (line 31) and then, after a second of delay in which he does not provide additional explanation, by asking him to specify what it is that he has to do (line 33). With this, she treats the issue as being unable to interpret his declining her request for help.


(8b)
31 SF: Hai da fare?
   have-2SG to do-INF
   **You have stuff to do?**
32     (1.1)
33 SF: Cos'hai da fare.
   what=have-2SG to do-INF
   **What do you have to do?**
34 FR: Non mi ricordo.qualcosa.
   not RFL remember-1SG something
   **I don't remember.something.**
35     (0.8)
36 SF: ((snorts))
37     (0.3)
38 SF: In realtà é una scusa per
   in reality be.3SG an excuse for
   **It's actually an excuse**
39     non venire,
   not come-INF
   **not to come**
40     (5.2)


In response to Sofia's questioning, Furio comes up short again and Sofia reproaches him further, snorting and then labeling his response as an excuse to not come. This last point highlights Furio's moral deficiency as rooted not in his declination *per se* but in his failure to make it comprehensible—specifically, to offer an adequate reason for his unwillingness.

All the apparently deviant cases in our collection where an accounted-for departure is nonetheless met with a reproach (*n* = 7/34) feature an account that fails along one or both of the lines just discussed. The most common failed account is a version of “I don't want to.” Although technically an account for refusing or declining an action, it is *internal* rather than *external*.

These findings are consistent with prior research on the “no fault” quality of typical accounts, which marshal interactants' knowledge of their own constraints to mitigate potential disaffiliation generated by departures (Heritage, 1984, p. 270–272). At the same time, our findings underscore the importance of externality and specificity to successful accounts, further indicating the degree to which mutual comprehensibility is entangled with moral positioning, and suggesting limits to what actors can legitimately assert regarding their own circumstances. Finally, our findings suggest limits to actors' freedom in developing *post-hoc* explanations. While the account need not correspond to the actor's “real” reasons for an action (Garfinkel, 1967; Vaisey, 2009), the ability to produce an acceptable account for problematic conduct depends on articulating external and specific reasons in the moment.

At this juncture, we have argued that the moral imperative to maintain action comprehensibility in social interaction explains when and how interactants police others' conduct and specifically whether or not violations are reproached. A question that remains from the existing literature, however, is how are reproaches designed and specifically when are they stronger or weaker. On the one hand, theories that view morality in social conduct as primarily driven by violations and their severity suggest that more severe violations should receive more severe reproaches (Tavory, 2011; Stets and Carter, 2012). On the other hand, theories that view morality in social conduct as primarily concerned with maintaining intelligibility according to standardized procedures suggest that the relative strength of a reproach should be less about the violation itself and more about its comprehensibility within a larger context (Garfinkel, 1967; Heritage, 1984).

### The persistence of the violator

Before we directly consider what conditions the design of reproaches, let us return to Table 1, where we summarized the types of reproaches found in the data. The four reproach types we identified are ordered alternatives, with *pursuits* being the weakest type of reproach and *sanctions* being the strongest. Several forms of evidence support this. First, when participants shift from one type of reproach to another (e.g., when a first reproach is not taken up by the violator), they consistently move upward. This is typical of the organization of ordered interactional practices, such as those used to initiate repair on problems of hearing or understanding (Schegloff et al., 1977). Repair initiation practices (e.g., “Huh?”, “Who?”, “You mean Susan?”) are ordered in terms of their power to locate and identify trouble sources (e.g., the whole prior turn vs. a particular person reference contained in it). As participants shift from one repair initiator to another, they use increasingly strong practices. We observe a similar pattern in the use of reproach practices in our data.

The ordering of reproach types is further supported by the degree to which they expose a violation. For instance, pursuits nudge the violator to provide the rule-conforming response, treating it as at least plausibly forthcoming. As such, pursuits do not highlight fault, thus helping participants preserve face (Goffman, 1967; Brown and Levinson, 1987). Consider (9), where a toddler is seated on the floor at the beginning of a family dinner. Her parents are watching from the table. In partial overlap, both parents observe the girl getting her hands dirty. Mom indirectly requests that Dad wash her hands (lines 3/4). Dad understands this, readily asking where the towel is (lines 5/6), which Mom ultimately answers (line 9). Our target pursuit comes a moment later when Dad has maintained his bodily position in his chair next to Mom at the table and, despite having just asked about the towel, has shown no indication of taking action.


(9) Liu
1  MOM: Get your [^fingers out-
2  DAD:   [^No no no not- HEY.
3  MOM: Her hands need to be washed.
4   =cuz she's gonna- she's gonna=
5  DAD: =Where's
6   [the towel (then)/(hon'),]
7  MOM: [put   her   fin]ger,= 
8  DAD: =Where's [the towel,
9  MOM:   [( ) on the ground,
10   (1.0)
11 MOM: [**Your tur:n.**
12 MOM: [((Body torque to dad))
13 DAD: °Hm?°
14 MOM: I'm eating. **Your turn.**
15  (I already...)


After Dad's failure to comply with Mom's request, there is a silence (line 10), at the end of which Mom pursues response by prompting him with “Your tur:n.” (line 11). This nudges Dad to comply without chastising, challenging, or otherwise halting the progress of the sequence. Mom pursues response again in line 14, and Dad ultimately complies.

Like pursuits, requests for confirmation also do not highlight fault. Although the action may thematize the problematic behavior through repetition (e.g., “You don't want a beverage?” in [5]), by asking violators to recommit to it, interlocutors create an opportunity for violators to revise their response. This action also works as an indirect way to solicit an account (Raymond and Stivers, 2016).

Beyond a request for confirmation, Extract (5) also features the third-strongest form of reproach, a challenge. Judy's “Why not?,” (line 11) can be understood as stronger than Gio's “You don't want a beverage?” because it no longer merely checks on whether Lance's position remains the same but specifically questions the comprehensibility of his conduct, exposing the lack of an explanation by directly soliciting one. Challenges no longer provide an opportunity for participants to revise their positions, instead treating the target behavior as deficient. Note too that, in the cases examined above, we consistently saw interlocutors reproach first with a request for confirmation and only subsequently with a challenge (5, 7, and 8). Finally, sanctions represent the strongest form of reproach because, with them, interlocutors no longer question but rather assert that the participant has done something wrong (e.g., [4]) or evaluate it as such (e.g., in [1]).

While the theory that interactants work to maintain the ongoing comprehensibility of their actions explains when violations are met with reproaches, it does not explain the strength with which interlocutors design those reproaches. When a violation lacks an adequate account, interlocutors can be found reproaching actors with all of the four practices. In what follows, we show that interlocutors issue stronger reproaches in cases where the rule-departing actor has *persisted* in a single violation over time, or engaged in multiple violations over the course of the interaction. This suggests that considerations of character and reputation are at least as important as the violation itself.

Consider (10), where Stephen Colbert is interviewing CNN news anchor Anderson Cooper about the then-prospective hearings on the January 6th storming of the US Capitol. Colbert asks Cooper first what he has heard they are planning to do or show, ending with the very broad “Do you know anything.” (lines 4/5). In response, Cooper offers a rather bland answer, saying “Uh::m I- I know they will be show:ing∧ some (.) videos,”. Colbert then asks a follow-up question about what the videos are. However, instead of offering a preferred “Yes” answer, Cooper provides a transformative answer, “Uh: I have an idea∧ of what they are?” (Stivers, 2022). Colbert continues for a third round with a follow-up question asking for Cooper's idea of what they are. Finally, on this iteration, Cooper stonewalls Colbert, declining to answer at all and giving no substantive account by saying “I- I wouldn't go into it?”.


(10) Colbert-Cooper: January 6th
1  CL: For the fall, .hh What- (.)
2   What- What have you heard
3   in your reporting that
4   they're planning tuh:: to
5   do, or show, do you know
6   anything.
7  CO: Uh::m I- I know they will
8   be show:ing^ some (.) videos,
9   um an[::d      ]
10 CL:   [>D'you know <] what
11     they are?
12 CO: Uh: I have an idea^ of what
13     they are?
14 CL: What's your idea °of what
15     they [are.°
16 CO:   [I- I- I wouldn't go
17     into it?
18     (0.4) ((audience laughter))
19 CO: Uh::_
20 CL: Why- Are your- You're a
21     newsman. Report.
22     [Report what you think.]
23 CO: [Huh  huh  I kn(h)ow   ]
24     (0.7)/((Audience laughter))
25 CL: it's gonna be.


Cooper's demurring elicits audience laughter and, in response, Colbert initiates what appears to be on its way to requesting an account with “Why-”, a challenge. He then quickly abandons that in favor of another form of question (“Are your-”), though this too is abandoned as Colbert then upgrades to a sanction “You're a newsman. Report. Report what you think.” This sanction is a strong first reproach, but it comes in an environment where Cooper has persisted in a series of uncooperative behaviors violating the preference for an answer, repeatedly evading Colbert's questions. Note that it is not only that Cooper fails to report but that this is also at odds with his role as a “newsman” suggesting that this moral failing is both the persistence with which he refuses to report and its inconsistency with his professional obligations.

Similarly, in (1), although it is the loudness of the radio that attracts the sanction, Brad has made incremental moves that compete with Ally starting with turning the radio on, then changing from music to news, and then increasing the volume to where it is difficult to hear her voice. Thus, the sanction comes not after the first time Brad competes with Ally's talk but after he has persisted in upping the degree of competition.

We see this again in (2), where Bruna initially acknowledges Ada's departure but then immediately sanctions her, not only for this instance but for what she claims is a pattern of continuing to request different questions or changes of the subject (lines 12–15 and 17–18). Thus, it is Ada's repeated behavior that is treated as the basis for her strong reproach. Finally, (4) shows another similar situation where Vic persists in answering a question addressed to Joe rather than to him. In this case, too, we see Mike coming in with a sanction: “Waidaminnit I didn' ask you,” after persistence has been established.

In contrast, weaker reproaches are found in contexts where the violator has not persisted in transgressions of some sort. For instance, in (5), Gio offered his housemates a “beverage” and Lance's departure is to decline the offer. This is initially reproached with a relatively weak request for confirmation. Only after this is rebuffed without providing an account does an interlocutor escalate to a challenge. Extract (7) is similar: Jan has not persisted in any interactional departure, and the initial reproach of his marked response is a request for confirmation. However, he then fails to explain the supposed “obviousness” of his dad's name being “Kramer” and instead insists that “He's a legend. He's a fuckin' legend”, thus treating the call for an account as not relevant. At this point, Jan has (like Lance in [6]) performed more than one transgression, leading Justin to escalate to a challenge.

In sum, we have shown that stronger reproaches are consistently found when actors persist in norm or preference departures. In contrast, when actors have only committed a single violation, they tend to receive weak reproaches. This pattern holds across the corpus: of the 15 norm violations that were reproached, 47% (*n* = 7) were reproached with pursuits, the weakest form of reproach, and 33% (*n* = 5) were reproached with sanctions, the strongest form of reproach. These proportions were not significantly different from those found in the set of 47 preference violations that were reproached, 34% of which (*n* = 16) were reproached with pursuits and 30% (*n* = 14) with sanctions (*p* = 0.791) (see Appendix B). Our empirical data thus support a theory of interactional policing that sees the moment-by-moment positioning of actors, rather than the avoidance of violations *per se*, as the primary moral obligation of interactants.

## Discussion

Norms and preferences are sustained by the ongoing activity of social actors, who work together to maintain and reproduce social order at the micro-level through interaction. While much of this work may be invisible, reproaches expose the fabric of the interaction order (Garfinkel, 1967; Goffman, 1983). Yet, despite treating social interaction as morally organized around shared norms and preferences, characterizations of the interaction order lack a theory of interactional policing. Empirically, our puzzle was to explain when and how reproaches are issued for interactional violations such as not responding to a question or declining an offer. Theoretically, our goal was to assess which of the two main versions of morality in the sociological literature best explains the dynamics of interactional policing.

We found that, within social interaction, the violation's import cannot be understood in isolation from its broader context. Our obligations to one another extend beyond mere obedience to discrete, isolated rules. Rather, we are responsible for upholding implicit moral contracts to maintain accountability, intersubjectivity, and to avoid persistent defiance of the social order. Making an interactional departure comprehensible through the provision of an account conditions the likelihood of being reproached. By accounting for departures, interactants uphold their positions as moral actors despite violating interactional rules, and interlocutors orient to this as the more relevant dimension of morality by not reproaching them. It is telling that, as interactants police others, the provision of accounts that are external and specific are sufficient to ward off reproaches. While they may seem trivial, accounts represent an orientation by speakers to others and their right to be able to make sense of speakers' ongoing behavior.

When it comes to how interactional policing is done, we offered a typology and showed that this too is conditioned not by the commission of norm or preference violations *per se* but by the immorality of persisting in those violations. When a violation is isolated, interlocutors tend to use weaker types of reproaches such as pursuits and requests for confirmation. When, on the other hand, a violator has repeatedly departed from interactional rules or persisted in their violation across multiple turns, interlocutors reproach them more strongly, with challenges or sanctions. These patterns suggest that it is the moral *positioning* of the violator more than the *nature* of the violating act that makes the difference for the policing of interactional rules.

A main implication of this study is that the interaction order rests on the linkage between temporal sequences, comprehensibility, and morality. Despite their portability, norms and preferences are not transsituational principles that operate in isolation from their larger settings. Rather, they are rendered salient in and through unfolding contexts. In determining whether to reproach a violation, interactants monitor one another, situating each new move in the context of what has come before and in anticipation of future developments. Within these contexts, norms allow us to identify what it is that is taking place as well as to regulate the appropriate next actions that should be taken (Heritage, 1984; Elster, 1989).^4^ Our ongoing need to render actions comprehensible by fitting them to the context points to the importance of accountability. People operate according to implicit expectations that others will conduct themselves as moral actors—conforming to norms and preferences but also maintaining the intersubjectivity of their social actions. Indeed, shared expectations about methods of interpretation constitute the basic preconditions for mutual intelligibility (Garfinkel, 1967; Levinson, 2000). Accounts, first and foremost, work to restore intersubjectivity by rendering the action intelligible in terms of shared expectations. However, merely rendering a violation mutually intelligible is not enough. To get out from under the moral obligation to comply, actors must successfully distance themselves from the violation. Accounts provide a vehicle through which such distancing can be accomplished, and interactants police one another for whether or not they perform this work.

Like reproaches, accounts exclude an action from the routine course of the social order, explicitly acknowledging its deviance and marking it as outside the bounds of normal behavior. In producing an account, actors demonstrate understanding of the violated rule, affirming competence in collective obligations regarding social behavior. They further claim that the relevant action is *atypical* and that they are not the sort of people who normally act in this way. This contention is facilitated by accounts that emphasize external constraints over subjective inclinations. Such assurances serve to protect relationships as well as reputations. Thus, when actors account for rejecting an offer, they work to localize the disaffiliative consequences of the rejection, marking it as detached from the larger relationship (e.g., I can't accept another helping of your cake because I'm on a diet, not because I don't like it). The kinds of accounts that we found to be most effective (those that identify external and specific constraints on the actor) facilitate the claim that the problematic action is an aberration. Such accounts thus reaffirm to the interlocutor that the actor shares a set of social commitments, rendering their behavior comprehensible and predictable in terms of shared heuristics (Elster, 1989; Gigerenzer, 2004). While our data cannot evaluate longitudinal claims, this argument is consistent with Scott and Lyman's (1968) suggestion that the efficacy of an account diminishes as an actor repeats it across different situations.

Our analysis further found that, in policing the interaction order, people consistently orient to the transgressor's trajectory of prior violations or compliance. Interlocutors often treat single errors or violations as worthy of a pass. Yet, when that transgressor has a track record—even if that is established over the course of minutes or seconds—interlocutors are more likely to reproach the transgression and to do so more strongly. Persisting in violating behavior, even if the violation is relatively minor, is treated as more egregious than an isolated, more severe violation. Thus, rather than focusing on the action itself, interlocutors treat the violator's prior conduct as key to the strength of their reproach. As noted in our Data and Methods section, our goal in this study was to have an adequate number of departures to explore the question of what types of departures are reproached and how reproaches are designed. Because we gathered a wide variety of departures and reproaches, we did not identify every departure that occurred in the data and can make no claims about their basic frequency in that sense. Future research should explore this, for example by systematically collecting all norm and preference departures within a particular data set.

Beyond the interaction order, our findings have implications for how sociologists of morality seek to uncover the normative frameworks toward which individuals orient themselves. Other social institutions can significantly differ from the interaction order and operate according to distinct moral frameworks. However, interaction provides the basic environment in which all social conduct takes place (Knorr-Cetina, 1988). In acting within and reproducing various institutional environments—whether law courts, classrooms, or medical encounters—actors selectively deploy resources drawn from the interaction order (Heritage and Clayman, 2011).

Given the privileged position of comprehensibility and persistence within interactional policing, sociologists of morality should be cautious about analyzing moral actions in isolation from the contexts in which they are enacted. In this regard, it is suggestive that reputations have wide-ranging consequences for how actors navigate and make sense of such diverse domains as the organization of interpersonal relationships (Goffman, 1963), the position of businesses within a market (Lange et al., 2011; Bartley and Child, 2014), and the relative status of state actors in international diplomacy (Rivera, 2008; Adler-Nissen, 2014; Stuart, 2018). Of course, there may, within some domains of social life, be violations for which no adequate account can be given, so that the nature of the violation is paramount. However, such a judgment should not be made *a priori*. Rather, careful examination should be given as to whether and to what extent comprehensibility and persistence are implicated in judgements about what violation has taken place.

## Data availability statement

The data analyzed in this study is subject to the following licenses/restrictions: Due to the ethics constraints, video recorded data cannot be directly shared. Requests to access these datasets should be directed to stivers@soc.ucla.edu.

## Ethics statement

The studies involving humans were approved by the University of California, Los Angeles. The studies were conducted in accordance with the local legislation and institutional requirements. Written informed consent for participation in this study was provided by the participants' legal guardians/next of kin. Written informed consent was obtained from the individual(s) for the publication of any potentially identifiable images or data included in this article.

## Author contributions

TS: Writing – review & editing, Writing – original draft, Methodology, Funding acquisition, Formal analysis, Data curation, Conceptualization. AC: Writing – review & editing, Writing – original draft, Methodology, Investigation, Formal analysis, Conceptualization. GR: Writing – review & editing, Writing – original draft, Methodology, Investigation, Funding acquisition, Formal analysis, Data curation, Conceptualization.

## Footnotes

^1^Some definitions of “sanction” also include positive reactions that reward behavior (Gibbs, 1966). However, the effect of positive and negative reactions is not symmetrical, suggesting that they should be analyzed separately; while rewards promote the participation of small groups of cooperators, negative sanctions tend to encourage broad compliance (Oliver, 1980). For this reason, in this paper we reserve the term “sanction” for punitive actions.

^2^Hassocks and ottomans are both footstools. Furniture stores state that ottomans can include storage but are otherwise the same.

^3^Note that the Italian construction used by Furio does not involve an object to the verb *do* (“fare”), so his account is phrased just that he has *to do* ∅ *this afternoon*, not specifying an activity.

^4^Norms facilitate the interpretive understanding of situations even when they are being violated. For example, it is the inversion of certain interactional norms, such as not speaking in overlap and not yelling, that makes an argument hearable *as* an argument (Heritage, 1984, p. 118; Goodwin and Goodwin, 1987).
