# Supplementary material for: To err is human but to persist is diabolical: Toward a theory of interactional policing
Source: Front Sociol. 2024 May 10;9:1369776. doi: 10.3389/fsoc.2024.1369776 (PMC11120954; doi:10.3389/fsoc.2024.1369776)
Supplement: Supplementary file 1 [file Data_Sheet_1.pdf]

Appendix A: Transcription Symbols

1. Temporal and sequential relationships

A. Overlapping or simultaneous talk is indicated in a variety of ways.

[Separate left square brackets, one above the other on two successive
[lines with utterances by different speakers, indicates a point of overlap onset, whether at the start of an utterance or later.

] Separate right square brackets, one above the other on two successive
] lines with utterances by different speakers indicates a point at which two overlapping utterances both end, where one ends while the other continues, or simultaneous moments in overlaps which continue.

= B. Equal signs ordinarily come in pairs -- one at the end of a line and another at the start of the next line or one shortly thereafter. They are used to indicate two things:

1) If the two lines connected by the equal signs are by the same speaker, then there was a single, continuous utterance with no break or pause, which was broken up in order to accommodate the placement of overlapping talk.

2) If the lines connected by two equal signs are by different speakers, then the second followed the first with no discernable silence between them, or was "latched" to it.

(0.5) C. Numbers in parentheses indicate silence, represented in tenths of a second; what is given here in the left margin indicates 1/2 second of silence. Silences may be marked either within an utterance or between utterances.

(.) D. A dot in parentheses indicates a "micropause," usually less than 200 milliseconds.

2. Aspects of speech delivery, including aspects of intonation.

A. The punctuation marks are not used grammatically, but to indicate intonation.

. The period indicates a falling, or final, intonation contour, not necessarily the end
? of a sentence. Similarly, a question mark indicates rising intonation, not
, necessarily a question, and a comma indicates slightly rising intonation, not
?, necessarily a clause boundary. A combined question mark and comma, which indicates a rise stronger than a comma but weaker than a question mark. An
_ underscore following a unit of talk indicates level intonation.

; The semicolon indicates that the intonation is equivocal between final and slightly rising

: : B. Colons are used to indicate the prolongation or stretching of the sound just preceding them. The more colons, the longer the stretching.

\- C. A hyphen after a word or part of a word indicates a cut-off or self-interruption.

word D. Underlining is used to indicate some form of stress or emphasis, either by increased loudness or higher pitch. The more underlining, the greater the emphasis.

° E. The degree sign indicates that the talk following it was markedly quiet or soft.
°° When there are two degree signs, the talk between them is markedly softer than the talk around it.

^ | G. The circumflex symbol (or upward arrow) indicates a rise in pitch. The pike (or downward arrow) indicates a drop in pitch.

> < H. The combination of "more than" and "less than" symbols indicates that the
< > talk between them is compressed or rushed. Used in the reverse order, they
< indicate that a stretch of talk is markedly slowed or drawn out. The "less than" symbol by
itself indicates that the immediately following talk is "jump-started," i.e., sounds like it starts with a rush.

hhh I. Hearable aspiration is shown where it occurs in the talk by the letter "h" -- the more h's, the more aspiration. The aspiration may represent breathing,
(hh) laughter, etc. If it occurs inside the boundaries of a word, it may be enclosed in parentheses in order to set it apart from the sounds of the word. If the aspiration is
.hh an inhalation, it is shown with a period before it.

J. A number sign indicates gravelly voice quality on the sound(s) that follow or that are between two number signs.

£ K. The British pound sign indicates “smile voice”.

3. Other markings.

(( )) A. Double parentheses are used to mark transcriber's descriptions of events, rather than representations of them. Thus ((cough)), ((sniff)), ((telephone rings)), ((footsteps)), ((whispered)), ((pause)) and the like.

(word) B. When all or part of an utterance is in parentheses, or the speaker identification is, this indicates uncertainty on the transcriber's part, but represents a likely possibility.

( ) Empty parentheses indicate that something is being said, but no hearing (or, in some cases, speaker identification) can be achieved.

Appendix B: Statistical analyses

Statistical analyses were conducted in RStudio (RStudio Team, 2022) with R 4.1.3 (R Core Team, 2022). We used the packages *lme4* 1.1-30 (Bates et al., 2015) and *ordinal* 2019.12-10 (Christensen, 2019) to fit generalized linear mixed models (Baayen, Davidson, and Bates, 2008) with the functions *glmer* for logistic regression and *clmm* (cumulative link mixed model) for ordinal logistic regression. Mixed-effects models allowed us to capture dependencies within the data at the level of interaction episodes (random effect) while testing the main predictor of interest (fixed effect). Statistically significant fixed effects were further tested by comparing the model to a null model with only the random effect (Forstmeier and Schielzeth, 2011) using a likelihood ratio test (R function *anova*).

(1) *Modeling reproaches as predicted by whether an account was provided at the time of the violation*

**Account at the time of the violation**	**Proportion (*n*) of violations reproached**	***N* observations**
Provided	.21 (7)	34
Not provided	.80 (55)	69

We fit a mixed-effects logistic regression model with the issuing of a *reproach* as the dependent variable (reproach not issued vs. issued); the provision of an *account* at the time of the violation as a fixed effect (account provided vs. not provided); and *interaction* as a random effect (intercept). The model, summarized below, yielded a statistically significant effect of not providing an *account* on the likelihood of a *reproach* being issued (OR 15.6, 95% CI 3.67–64.74, $p < .001$). We then compared this model (AIC 110.18, logLik -52.089) to a null model with only the random effect of *interaction* (AIC 142.42, logLik -69.208), resulting in a statistically significant difference ($\chi^2(1)$ 34.239, $p < .001$).

**Fixed effects**	**Log odds**	***SE***	***z***	***p***
(intercept)	-1.372	0.571	-2.402	.016 *
account: not provided	2.747	0.726	3.784	.000 ***
**Random effects**	**Variance**	***SD***		
interaction	0.047	0.217		
(observations = 103; *interactions* = 44)				

(2) *Modeling reproaches as predicted by the nature of the violation (norm or preference)*

**Nature of violation**	**Proportion (*n*) of violations reproached**	***N* observations**
Norm	.75 (15)	20

Preference	.57 (47)	83

We fit a mixed-effects logistic regression model with the issuing of a *reproach* as the dependent variable (reproach issued vs. not issued); the *nature* of the violation as a fixed effect (norm vs. preference); and *interaction* as a random effect (intercept). The model, summarized below, did not yield a statistically significant difference.

**Fixed effects**	**Log odds**	***SE***	***z***	***p***
(intercept)	-1.105	0.532	-2.077	.038 *
nature of the violation: preference	0.860	0.588	1.463	.144
**Random effects**	**Variance**	***SD***		
interaction	0.116	0.341		
(observations = 103; *interactions* = 44)				

(3) ***Modeling the strength of reproaches as predicted by the nature of the violation***

**Nature of the violation**	**Proportion (*n*) of pursuits**	**Proportion (*n*) of requests for confirmation**	**Proportion (*n*) of challenges**	**Proportion (*n*) of sanctions**	***N* observations**
Norm	.47 (7)	.0 (0)	.20 (3)	.33 (5)	15
Preference	.34 (16)	.08 (4)	.28 (13)	.30 (14)	47

We fit a mixed-effects ordinal regression model with the *strength* of reproaches as the dependent variable (pursuit < request for confirmation < challenge < sanction); the *nature* of the violation (norm vs. preference) as a fixed effect; and *interaction* as a random effect (intercept). The model, summarized below, did not yield a statistically significant effect.

**Fixed effects**	**Log odds**	***SE***	***z***	***p***
nature of the violation: preference	0.1541	0.582	0.265	.791
**Random effects**	**Variance**	***SD***		
interaction	0.092	0.304		
(observations = 62; *interactions* = 29)				

**Threshold coefficients**	**Log odds**	***SE***	***z***	***p***

pursuit | request for conf.	-0.414	0.535	-0.773	.440
request for conf. | challenge	-0.137	0.529	-0.259	.795
challenge | sanction	0.961	0.536	1.795	.073

REFERENCES

Baayen, R. H., Davidson, D. J., and Bates, D. M. (2008). Mixed-Effects Modeling with Crossed Random Effects for Subjects and Items. *Journal of Memory and Language,* 59 (4), 390–412. https://doi.org/10.1016/j.jml.2007.12.005.

Bates, D., Mächler, M., Bolker, B., and Walker, S. (2015). "Fitting Linear Mixed-Effects Models Using Lme4." *Journal of Statistical Software* 67(1), 1-48. https://doi.org/10.18637/jss.v067.i01.

Christensen, R. H. B (2019). Ordinal — Regression Models for Ordinal Data." https://CRAN.R-project.org/package=ordinal.

Forstmeier, W., and Schielzeth, H. (2011). "Cryptic Multiple Hypotheses Testing in Linear Models: Overestimated Effect Sizes and the Winner's Curse." Behavioral Ecology and Sociobiology, 65(1), 47–55. https://doi.org/10.1007/s00265-010-1038-5.

R Core Team (2022). R: A Language and Environment for Statistical Computing. Vienna, Austria: R Foundation for Statistical Computing. https://www.R-project.org/.

RStudio Team (2022). RStudio: Integrated Development Environment for R. Boston: RStudio, PBC. http://www.rstudio.com/.
